# Parental Genome Dosage Imbalance Deregulates Imprinting in *Arabidopsis*

**DOI:** 10.1371/journal.pgen.1000885

**Published:** 2010-03-19

**Authors:** Pauline E. Jullien, Frédéric Berger

**Affiliations:** 1 Temasek Life Sciences Laboratory, National University of Singapore, Singapore; 2 Zentrum für Molekularbiologie der Pflanzen, Entwicklungsgenetik, Universität Tübingen, Tübingen, Germany; 3 Department of Biological Sciences, National University of Singapore, Singapore; The Salk Institute for Biological Studies, United States of America

## Abstract

In mammals and in plants, parental genome dosage imbalance deregulates embryo growth and might be involved in reproductive isolation between emerging new species. Increased dosage of maternal genomes represses growth while an increased dosage of paternal genomes has the opposite effect. These observations led to the discovery of imprinted genes, which are expressed by a single parental allele. It was further proposed in the frame of the parental conflict theory that parental genome imbalances are directly mirrored by antagonistic regulations of imprinted genes encoding maternal growth inhibitors and paternal growth enhancers. However these hypotheses were never tested directly. Here, we investigated the effect of parental genome imbalance on the expression of *Arabidopsis* imprinted genes *FERTILIZATION INDEPENDENT SEED2* (*FIS2*) and *FLOWERING WAGENINGEN* (*FWA*) controlled by DNA methylation, and *MEDEA* (*MEA*) and *PHERES1* (*PHE1*) controlled by histone methylation. Genome dosage imbalance deregulated the expression of *FIS2* and *PHE1* in an antagonistic manner. In addition increased dosage of inactive alleles caused a loss of imprinting of *FIS2* and *MEA*. Although FIS2 controls histone methylation, which represses *MEA* and *PHE1* expression, the changes of *PHE1* and *MEA* expression could not be fully accounted for by the corresponding fluctuations of *FIS2* expression. Our results show that parental genome dosage imbalance deregulates imprinting using mechanisms, which are independent from known regulators of imprinting. The complexity of the network of regulations between expressed and silenced alleles of imprinted genes activated in response to parental dosage imbalance does not support simple models derived from the parental conflict hypothesis.

## Author Summary

In mammals and plants, imprinted genes are expressed preferentially by the copy inherited from either the mother or the father. In plants genome dosage is easily manipulated using tetraploid plants that contain twice the genome dosage of the natural diploid plants. The increased maternal dosage reduces seed size while increased paternal dosage has the opposite effect. It was further proposed that parental genomic imbalances are directly mirrored by antagonistic regulations of imprinted genes encoding maternal growth inhibitors and paternal growth enhancers. However these hypotheses were never tested directly. We measured the expression of imprinted genes and their regulators, in crosses between diploid and tetraploid *Arabidopsis* plants. Surprisingly, parental dosage imbalance affected each imprinted gene in a different manner and the imprinted status was also affected. Our results point to a relationship between imprinting and dosage imbalance that is more complex than predicted.

## Introduction

In mammals and plants, mothers differentiate distinctive structures specialized in the transport of maternal nutrients to the embryo, the mammalian placenta and the plant seed endosperm [1]. Thus, unilateral maternal contribution of nutrients results in an imbalanced parental contribution to the offspring. Such imbalance has been considered, in the frame of the kinship theory, as a potential cause for parental conflict of interest over allocation of resources to embryos [2],[3]. This hypothesis has gained support in mammals and in plants from the effects of parental genome dosage imbalance on embryo growth in plants and animals [4]–[8]. These observations were followed by the discovery of imprinted genes expressed preferentially from one parental allele [1],[9],[10]. The parental conflict hypothesis, derived from the kinship theory, proposes a competition over resource allocation to the embryo between imprinted genes encoding paternally expressed enhancers of embryo growth (PEGs) and maternally expressed inhibitors of embryo growth (MIGs) [11]. This hypothesis further suggests that increased maternal genome dosage results in increased levels of MIGs transcripts causing reduced embryo growth. A symmetrical increased paternal genome dosage is expected to result in increased levels of PEGs transcripts producing larger embryo. Although the parental conflict hypothesis was supported to a certain extent [2], [9], [10], [12]–[16], computational analyses on the origin of the selection of imprinting at the *MEA* locus did not lead to unequivocal support [17]–[19]. However, the response to dosage imbalanced is likely involved in deregulation of imprinted genes leading to sexual reproductive barriers [14] as suggested by studies involving *Arabidopsis* relatives [20],[21]. Although recent evidence suggested that a mutation causing the production of diploid male gametes deregulates imprinted gene expression when crossed to diploid wild type [22], the expression of imprinted genes in response to genome dosage imbalance in a wild type *Arabidopsis* background remained to be tested in order to provide experimental evidence for the parental conflict theory in plants.

Currently the regulation of five maternally expressed imprinted genes have been characterized in *Arabidopsis*, the Polycomb Group (PcG) gene *MEDEA* (*MEA*) [23], the gene *MATERNALLY EXPRESSED PAB C-TERMINAL* (*MPC*) [24], the PcG gene *FERTILIZATION INDEPENDENT SEED 2* (*FIS2*) [25], the transcription factor *FWA* [26], and the actin regulator *FORMIN5* [27]. The overall effect of loss-of-function of *MEA* and *FIS2* causes enhanced endosperm growth [28],[29] leading to the conclusion that these two genes represent potential MIGs as predicted by the parental conflict hypothesis. By contrast, *FORMIN5* loss of function leads to a reduction of endosperm growth and does not conform to the prediction of the parental conflict theory [27]. The transcription factor *PHERES1* (*PHE1*) is a paternally expressed imprinted gene in *Arabidopsis*, which could play a role as a PEG [30],[31]. Additional imprinted genes have been characterized in *Arabidopsis* [32] but their function remains to be determined.

Plant reproduction is initiated by a double fertilization event [33]. Two haploid sperms are delivered to the female gametes, the egg cell and the central cell. Fertilization of the haploid egg cell leads to embryogenesis. The second sperm cell fuses with the central cell producing the endosperm. The endosperm can be considered as an embryonic annex, which nurtures embryo development [10]. Parental imbalance of genome dosage in maize affects endosperm growth, which in turn influences embryo and seed growth [4],[8]. In *Arabidopsis*, increased maternal genome dosage in seeds resulting from crosses between ovules from tetraploid plants and pollen from diploid plants (4n_mat_×2n_pat_) leads to production of smaller endosperm, embryo and seed [5]. Reciprocal crosses (2n_mat_×4n_pat_) cause the opposite effect. These results have suggested that collectively increased dosage of the expressed maternal allele of MIGs reduces endosperm growth while increased dosage of the expressed paternal allele of PEGs increases endosperm growth [11].

Although it was assumed that parental dosage imbalances would be directly mirrored by variations in the expression of the PEGs and MEGs [2],[3],[11],[14], it became apparent that *MEA* and *PHE1* expression were regulated by *FIS2* [31], [34]–[36]. This cross-regulation between imprinted genes could thus impact on the expression levels of *MEA*, *FIS2*, *FWA* and *PHE1* in seeds resulting from interploid crosses. We performed quantitative RT-PCR to assess the expression of imprinted genes in endosperm produced by crosses between diploid and tetraploid plants and observed a global deregulation of expression levels of imprinted genes accompanied by an unexpected loss of parental imprinting for some genes. However the expression of known key regulators of imprinting were not affected. Our results suggest that parental dosage imbalance disrupts imprinting through interactions between imprinted genes and other unidentified regulators.

## Results/Discussion

### Increased paternal dosage causes silencing of FIS2 controlled by DNA methylation

**Figure 1:**
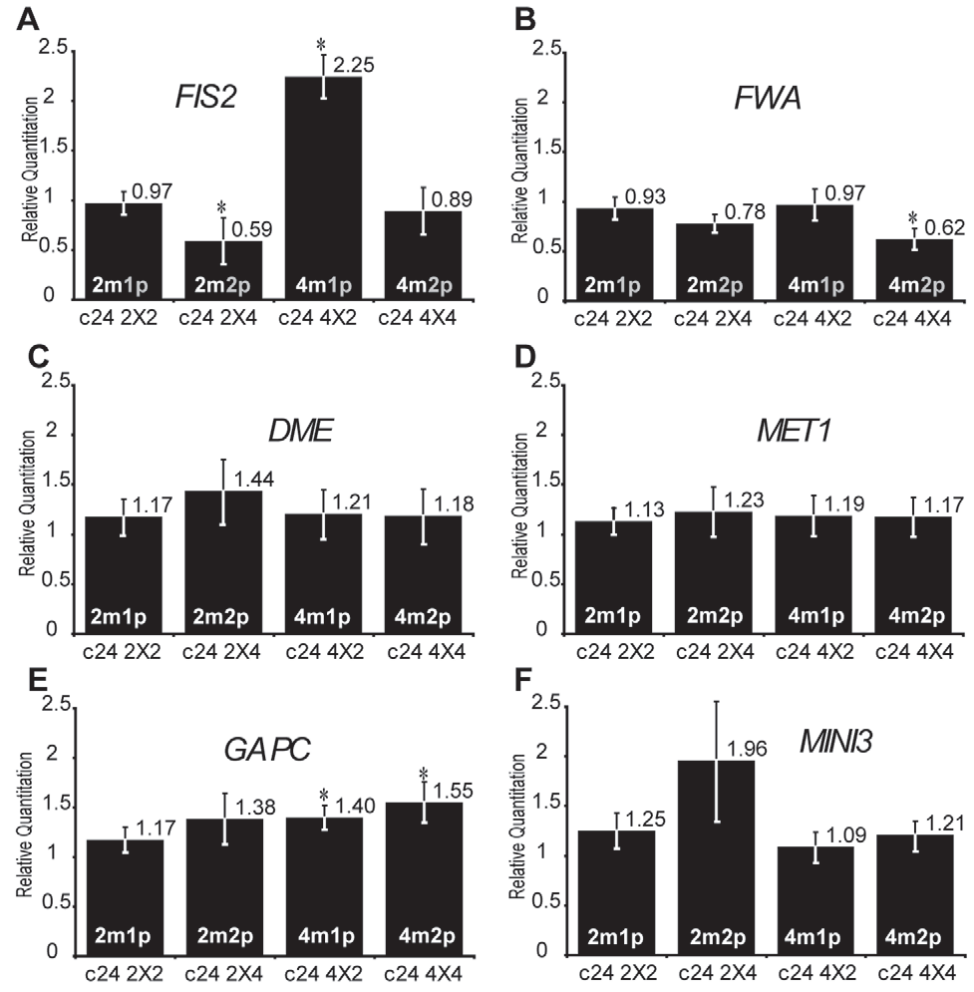
Effects of interploid crosses on the expression of DNA methylation-dependent imprinted genes. Quantitative PCR measurements of *FIS2* (A), *FWA* (B), *DME* (C), *MET1* (D), *GAPC* (E), and *MINI3* (F) mRNAs were performed on total mRNAs extracted from siliques produced by crosses between diploid and tetraploid parents (2 DAP, C24 ecotype). Each point represents the average RQ value obtained for four independent biological samples (values can be found in Table S1). Error bars represent the standard deviation. * represents p<0.05 of Student's *t*-test using C24 2X2 as a reference, All p values can be found in Table S2. The genome copy number in the endosperm is represented inside each bar (active copy in white, inactive copy in grey).

Increased maternal dosage is expected to increase the level of expression of the active maternal allele of *FIS2* and *FWA*. Conversely, the global level of expression of these genes should not be affected by an increased dosage of inactive paternal alleles. We used quantitative RT-PCR to investigate the effect of increased parental dosages in crosses between tetraploid and diploid plants. We measured the expression at 2 days after pollination (2DAP) when the imprinted genes studied are highly expressed and control the timing of endosperm development [28]. Between fertilization and 2 days after pollination the developmental pattern and size of endosperm size is not affected, [5] suggesting that we could observe direct consequences of parental genome imbalances. We performed the experiments in two genetic backgrounds C24 (Figure 1, Table S1) and Columbia (Col) (Figure S1, Table S3) and obtained similar results. We observed variations of higher amplitude in Col background and conservatively took into account only significant changes observed in both backgrounds and supported by statistical tests (Tables S2 and S4). We investigated the effects of genome dosage imbalance in non-imprinted genes expressed in the seed as *GAPC* or more specifically in endosperm as *MINI3* [37] and did not observe significant fluctuation of their expression levels (Figure 1E and 1F and Figure S1E and S1F). Similarly we did not observe significant changes in the expression of the two essential regulators of imprinting encoding DEMETER (DME) [38] and the DNA METHYLTRANSFERASE1 (MET1) [25],[26],[39] (Figure 1C and 1D and Figure S1C and S1D). The expression of these two genes is also not imprinted (data not shown). Our measurements thus indicated that parental genome dosage imbalance did not affect transcription globally and did not affect regulators of DNA methylation, which control imprinting.

We tested the effect of genome dosage imbalance on maternally expressed imprinted genes *FIS2* and *FWA,* which are silenced by DNA methylation [25],[26]. We observed hardly any changes in levels of *FWA* expression (Figure 1B and Figure S1B). By contrast to *FWA, FIS2* expression levels were very sensitive to parental genome imbalance. Levels of expression in self-fertilized 2n and 4n crosses were comparable (Figure 1A). As expected, supplementary doses of active maternal *FIS2* alleles produced by (4n_mat_×2n_pat_) crosses increased *FIS2* mRNA levels in endosperm (Figure 1A). Surprisingly, although (2n_mat_×4n_pat_) crosses did not change the dosage of transcriptionaly active maternal *FIS2* alleles, *FIS2* expression was reduced in comparison to seeds produced by self-fertilized diploid plants. (Figure 1A). We obtained similar results in Columbia background (Figure S1A). A similar decrease of *FIS2* expression was reported from 3 to 5 DAP in Landsberg *erecta* background using the meiotic *jason* (*jas*) mutant, which produces a proportion of diploid pollen [22]. Increased paternal dosage also reduced expression of the transcriptional reporter *pFIS2-GUS*, which contains the transcriptional regulatory cis-elements required for imprinting regulation [25],[40] (Figure S2). Thus, increased paternal genome dosage down-regulates *FIS2* expression irrespective of its genomic context.

Trans-silencing in *Arabidopsis* and maize [41]–[44] has been associated with the production of small interfering RNAs [45]. However, non-coding RNAs have not been shown to affect *FIS2* expression and the down-regulation of *FIS2* expression in response to increased dosage of inactive paternal alleles likely result from a distinct mechanism. We propose that the unexpected silencing of the maternal alleles of *FIS2* in endosperm produced by (2n_mat_×4n_pat_) crosses could originate from increased paternal dosage of a paternally expressed imprinted inhibitor of *FIS2* or from the activity of yet unidentified cis-elements.

### Interploid crosses cause deregulation of imprinting

**Figure 2:**
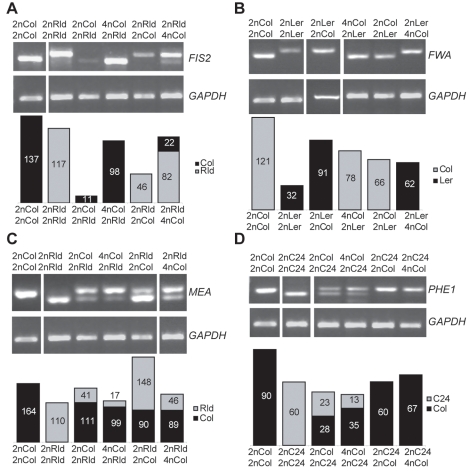
Effect of interploid crosses on imprinted status. (A) The imprinting of *FIS2* is detected by a size polymorphism between the strains RLD and Columbia (Col). The parent indicated at the top is the mother. (B) The imprinting of *FWA* is detected by a restriction polymorphism between the strains Ler and Col. (C) The imprinting of *MEA* is detected by a restriction polymorphism between the strains RLD and Col. (D) The imprinting of *PHE1* is detected by a restriction polymorphism between the strains C24 and Col. *GAPDH* is used as control. The band quantification is represented below each gel as a percentage of the *GAPDH* band intensity.

We assessed the imprinted status of *FIS2* and *FWA* in interploid crosses. Both genes remained strictly maternally expressed in (4n_mat_×2n_pat_) crosses (Figure 2A and 2B). This indicated that the increased dosage of active maternal alleles was directly responsible for the increased expression levels of *FIS2*. The opposite (2n_mat_×4n_pat_) crosses did not affect the *FWA* imprinting status (Figure 2B) but caused an unexpected paternal expression of *FIS2*, resulting in the loss of *FIS2* imprinting (Figure 2A). Loss of *FIS2* imprinting was not restricted to RLD 2n X Col 4n crosses as it also occurred in crosses using Ler 2n and C24 2n (data not shown). We thus concluded that increased paternal dosage decreases the overall expression of *FIS2* while both parental alleles become expressed. Such rather paradoxical effect is difficult to interpret. A negative interaction between MET1 activity, which maintains silencing marks and the trans-silencing mechanisms activated by the increased dosage of silenced paternal allele may cause removal of the silencing marks on the paternal allele of *FIS2.* Alternatively in response to reduced *FIS2* expression, a transcriptional activator of *FIS2* might be over-expressed and overcome silencing of the paternal allele.

We tested whether parental genome dosage imbalance would also deregulate imprinting of the genes *MEA* and *PHE1. MEA* was predominantly expressed from the maternal allele in (4n_mat_×2n_pat_) crosses as in control diploid crosses (Figure 2C). Surprisingly in (2n_mat_×4n_pat_) crosses the expression from the maternal allele decreased causing a predominant paternal expression of *MEA* leading to an apparent inversion of *MEA* imprinted expression (Figure 2C). *PHE1* imprinted status was not altered in response to paternal genome increase (Figure 2D). However *PHE1* imprinting is hardly observed in crosses between Col females and C24 males [31], and we could not assess the effect of increased maternal dosage on *PHE1* imprint (Figure 2D). In conclusion we observed that at least two out of four genes studied lost imprinting as a result of dosage imbalance. These results suggest that increasing the dosage of the silenced allele of an imprinted gene causes the removal of the imprinting marks on the silenced allele.

### Dosage imbalances effect on MEA and PHE1 indicate crosstalk between imprinted gene regulations

**Figure 3:**
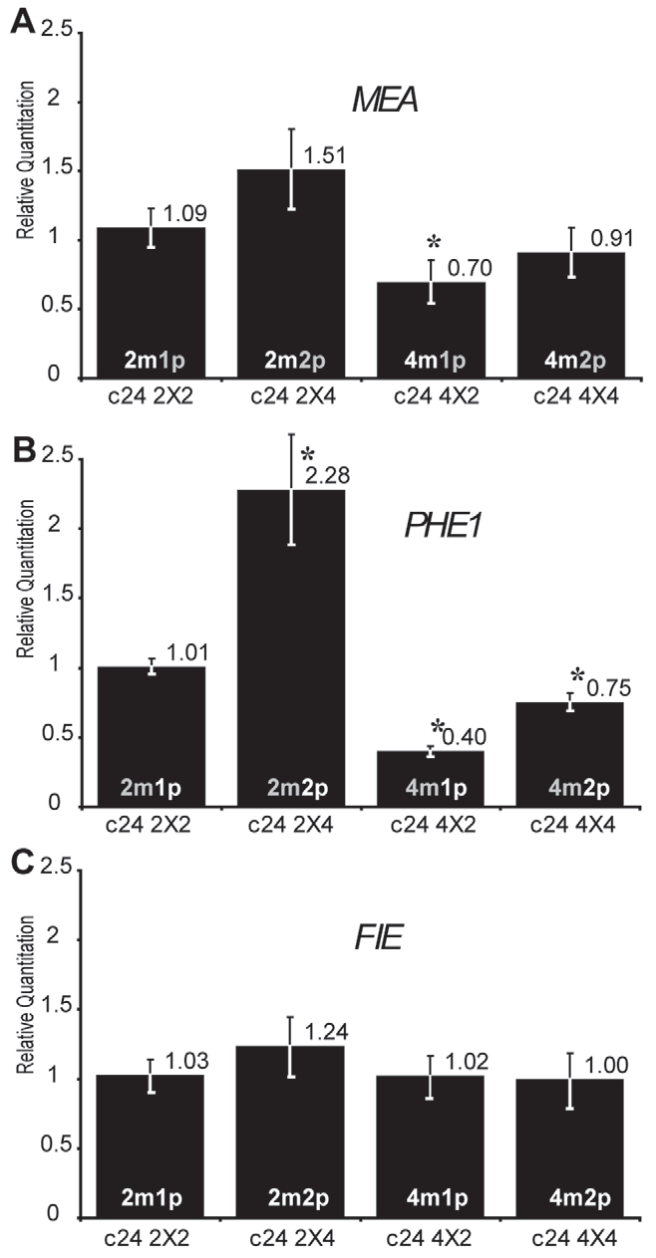
Effects of interploid crosses on the expression of genes imprinted by Polycomb group activity. Quantitative PCR measurements of *MEA* (A), *PHE1* (B), and *FIE* (C) mRNAs were performed on total mRNAs extracted from siliques produced by crosses between diploid and tetraploid parents (2 DAP, C24 ecotype). Each point represents the average RQ value obtained for four independent biological samples (values can be found in Table S1). Error bars represent the standard deviation. * represents p<0.05 of Student's *t*-test using C24 2X2 as a reference, p values can be found in Table S2. The genome copy number in the endosperm is represented inside each bar (active copy in white, inactive copy in grey).

We further tested the effect of dosage imbalance on the maternally expressed imprinted gene *MEA*, which is silenced by PcG mediated H3K27 trimethylation of its paternal allele [35],[36]. Although *MEA* is maternally expressed, *MEA* expression was repressed when the maternal genome dosage increased in (4n_mat_×2n_pat_) crosses in C24 background (Figure 3A, Table S1 and Table S2) but not in Col background (Figure S3A, Table S3 and Table S4). A modest increased *MEA* expression in response to increased paternal dosage was observed in Col background (Figure S3A). A mild increase was also observed at 1 DAP in Ler background using *jas* mutant mimicking (2n_mat_×4n_pat_) crosses [22].

**Figure 4:**
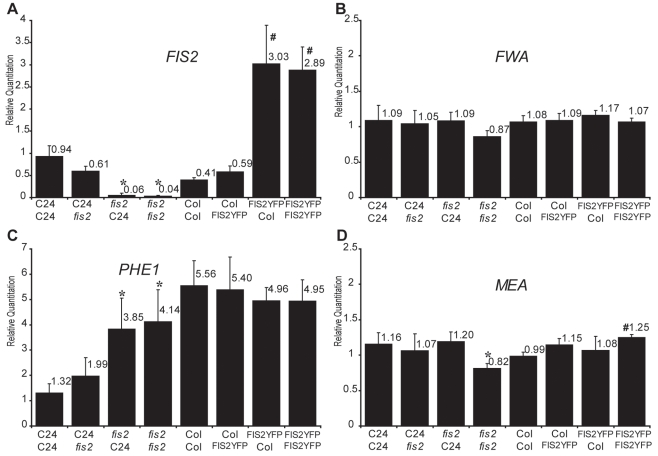
Effects of *FIS2* mRNA levels on the expression of imprinted genes. Quantitative PCR measurements of *FIS2* (A), *FWA* (B), *PHE1* (C), and *MEA* (D). mRNAs were performed on total mRNAs extracted from siliques produced by crosses between C24 and *fis2-6* parents (2 DAP, C24 ecotype) to test for the effect of *FIS2* down-regulation and between Col and FIS2YFP (2DAP, Col ecotype) to test the effect of increased *FIS2* expression. Each point represents the average RQ value obtained for three independent biological samples. Error bars represent the standard deviation. * represents p<0.05 of Student's *t*-test using C24 2X2 as a reference, # represents p<0.05 of Student's *t*-test using Col 2X2 as a reference, p values can be found in Table S5.

Parental dosage imbalances strongly perturbed *PHE1* expression following the trends exhibited by *MEA* expression levels (Figure 3B and Figure S3B) although *PHE1* is paternally expressed. A strong increase of *PHE1* expression was also observed after 3 DAP in Ler background using *jas* mutant as pollen donor [22]. These results could be explained by the common regulation of *MEA* and *PHE1* expression by the PcG complex, which contains *FIS2* and *MEA* and is active in endosperm [35],[36],[46]. *FIS2* encodes a *Suppressor of zeste 12* (*Su(z)12*) Polycomb group subunit [47]. Since the two other members of the *Su(z)12* family are not expressed in *Arabidopsis* endosperm [40] the reduction of *FIS2* expression levels in (2n_mat_×4n_pat_) crosses could become limiting for Polycomb group activity, leading to increased expression of *MEA* and *PHE1*. This effect would also be directly responsible for the inversion of *MEA* imprinting (Figure 3C) as previously shown for the effect of reduced Polycomb activity in loss of function mutants for *FERTILIZATION INDEPENDENT ENDOSPERM* (*FIE*) [35],[36]. We verified that parental dosage imbalance and tetraploidy do not affect *FIE* expression levels (Figure 3C and Figure S3C). We further tested whether alterations of *FIS2* expression would mimic the effects observed on *MEA* and *PHE1* in response to parental genome dosage imbalance. We used the loss of function allele *fis2-6* to decrease the levels of *FIS2* expression and a transgenic line expressing a complementing FIS2-YFP fusion protein to increase the levels of *FIS2* expression [48] (Figure 4, Table S5). Manipulating *FIS2* mRNA levels (Figure 4A) did not affect *FWA* expression (Figure 4B). We did not observe any effect of increased *FIS2* levels on *PHE1* expression levels (Figure 4C). However we observed that decreased *FIS2* expression causes increased *PHE1* expression but did not affect *MEA* expression. Despite the fact that *MEA* and *PHE1* are over-expressed in response to decreased FIS PcG activity [36], [46].

We conclude that increased *FIS2* expression caused by maternal genome dosage increase is not directly responsible for the decreased expression of *MEA* and *PHE1*. By contrast, paternal genome dosage causes an unexpected decrease of *FIS2* expression, which in turn could directly or indirectly increase *PHE1* expression. As an alternative explanation, increased dosage of *PHE1* copy number might rather directly increase *PHE1* expression in response to paternal dosage increase.

### Conclusions

Reciprocal changes of parental dosage do not cause the symmetrical variations of expression of imprinted genes predicted by previous studies. A similar complex phenomenon was observed in mammals [48]–[52]. However parthenogenetic embryos used in mice to investigate parental dosage imbalance do not allow a direct test for the interactions between the paternal and maternal allele. In addition parthenogenotes are produced via complex in vitro manipulations and other factors may perturb silencing at imprinted loci. In plants dosage imbalances are created in vivo in undisturbed reproductive tissues and their consequences are unlikely to reflect the consequence of experimental manipulations. In response to parental imbalance we observed unexpected non-symmetrical deregulation of the expression of imprinted genes coupled with a loss of imprinting in two out of four imprinted genes studied. The modulation of seed size by dosage imbalance does not result in variations of PEGs and MIGs expression parallel to variation of the dosage of the respective parental genome. In addition, the mode of perturbation may vary during later development stages in endosperms produced by crosses involving *jas* mutant that produces a fraction of diploid pollen [22].

After 6 DAP, paternal excess dosage causes endosperm developmental defects similar to loss of FIS complex activity [5]. The fact that *MEA* ectopic expression rescues late endosperm developmental defects in crosses with *jas* pollen [22] suggests that the late perturbations of imprinted genes expression in response to *jas* pollination may result rather from an indirect deregulation of endosperm developmental timing caused by loss of FIS activity [28]. At early stages of endosperm development we do not observe a strong link between *FIS2* expression and the perturbation of *MEA* and *PHE1* expression. Thus our data do not support that the FIS PcG complex directly deregulates imprinted genes expression in response to dosage imbalance a couple of days after fertilization. In addition parental genome dosage imbalance does not affect expression of *FIE,* the essential component of the FIS PcG complex. Parental genome dosage imbalance does not affect expression of the regulators of DNA methylation MET1 and DME. Hence, parental dosage imbalance does not directly affect the known major controls of imprinting. Nevertheless, we propose that parental dosage imbalance deregulates *FIS2* and *MEA* expression, which causes late endosperm developmental defects including over-proliferation and ectopic expression of PHE1. A similar phenotype has been observed in crosses between *A. thaliana* and *A. arenosa.* Such deregulation compromise seed viability and likely contribute to species isolation mechanisms involving tetraploidization [21], [43], [53].

We do not currently understand the mechanisms that cause the immediate response to dosage imbalance and deregulation of *FIS2*, *MEA* and *PHE1* expression. Such mechanisms could involve other controls of DNA methylation [54] or small non-coding RNAs inherited maternally [55] or paternally [56]. The apparent parental conflict linked to imprinting in plants and in mammals likely results from a complex series of non-symmetrical regulations during zygotic development. Nevertheless these mechanisms could involve imprinted regulators controlled in a dosage dependent manner predicted by the kinship theory [2], [57].

## Materials and Methods

### Plant lines

The wild-type control lines C24, Col, Ler, and RLD were obtained from the ABRC stock center. The tetraploid lines in C24 and in Col ecotypes were kindly provided by Rod Scott [5] and Luca Comai [58]. The reporter line *pFIS2-GUS* (C24 accession) was kindly provided by Abed Chaudhury [40]. FIS2YFP line was kindly provided by Ramin Yadegari [48] and *fis2-6* was previously identified in our laboratory [59].

### Allele-specific RT–PCR and quantitative real-time RT–PCR

Siliques two days after pollination (2DAP) were collected from *Arabidopsis* plants and frozen in liquid nitrogen. Total RNAs were extracted using the RNeasy mini kit (Qiagen). After DNAse treatment using DNase free kit (Ambion), RNAs were reverse-transcribed using Stratascript RT kit (Stratagene).

Allele-specific RT-PCR reactions were performed as previously described [23],[25],[26],[60]. Band quantification was performed using the ImageJ software (http://rsbweb.nih.gov/ij/).

Real-time PCR assays were performed using a PCR Master Mix (Applied Biosystems, Foster City, CA). One µl of RT product was used to perform each PCR reaction. Amplification reaction was carried out using specific primers at a concentration of 0.5 mM in a 10 µl reaction. Sequence of specific primer pairs can be found in Table S6. The specificity of the amplification product was determined by performing a dissociation curve analysis. PCR efficiency was determined using the LinReg program [61]. The PCR reaction and quantitative measurements were achieved with 7900HT Fast Real-Time PCR System (Applied Biosystems, Foster City, CA). Thermal cycling parameters were 2 min at 50°C, 10 min at 95°C and 50 cycles of 15 sec at 95°C, 60 sec at 60°C. We performed four biological replicates, with three technical replicates for each sample. For each PCR reaction the ΔCt was calculated using *ACT11* gene as endogenous control except for Figure 4 were *FIE* was used as endogenous control. Relative Quantitation values (RQ) were calculated using the 2^−ΔΔCt^ method (RQ = 2^−ΔΔCt^) [62]. Values given in Figure 1, Figure 3, Figure 4, Figure S1 and Figure S3 represent the average of RQ values obtained for four or three biological replicates for each point and the error bars represent the standard deviation of the biological replicates. Tables of RQ values used to make the graphs can be found in Table S1 for C24 accession and Table S3 for Col accession.

## Supporting Information

Figure S1 Effects of interploid crosses on the expression of DNA methylation dependent imprinted genes. (A-D) Quantitative PCR measurements of *FIS2* (A), *FWA* (B), *DME* (C), *MET1* (D), *GAPC* (E), and *MINI3* (F) mRNAs were performed on total mRNAs extracted from siliques produced by crosses between diploid and tetraploid parents (2 DAP, Col ecotype). Each point represents the average RQ value obtained for four independent biological samples (Table S3). Error bars represent the standard deviation. * represents p<0.05 of t-test using Col 2X2 as a reference, p values can be found in Table S4.
(0.89 MB TIF)

Figure S2 Effects of interploid crosses on the expression *FIS2* transgenes. Effect of an increased maternal dosage on expression of transcriptional reporter *pFIS2-GUS* expression at 1.5 DAP. Staining was stopped before signal saturation and three classes of seeds were distinguished on the basis of the intensity of signal. The percentage of each class in crosses between ovules of the marker line and wild-type pollen from diploid or tetraploid plants is indicated below each corresponding micrograph. Scale bars correspond to 25 µm.
(2.58 MB TIF)

Figure S3 Effects of interploid crosses on the expression of genes imprinted by Polycomb group activity. Quantitative PCR measurements of *MEA* (A), *PHE1* (B), and *FIE* (C) mRNAs were performed on total mRNAs extracted from siliques produced by crosses between diploid and tetraploid parents (2 DAP, Col ecotype). Each point represents the average RQ value obtained for four independent biological samples (Table S3). Error bars represent the standard deviation. * represents p<0.05 of t-test using Col2X2 as a reference, p values can be found in Table S4.
(0.75 MB TIF)

Table S1 RQ value of the C24 experiment after normalisation with *Act11*. C24 2nX2n sample 1 was normalised to 1. Each RQ value in this table corresponds to the average RQ value of 3 technical replicates.
(1.32 MB TIF)

Table S2 Probability values obtained after a student's t-test on C24 sets of crosses from Figure 1 and Figure 3. Two samples are significantly different when p<0.05.
(1.31 MB TIF)

Table S3 RQ value of the Columbia experiment after normalisation with *Act11*. Col 2nX2n sample 1 was normalised to 1. Each RQ value in this table corresponds to the average RQ value of 3 technical replicates.
(1.34 MB TIF)

Table S4 Probability values obtained after a student's t-test on Col sets of crosses from Figure S1 and Figure S3. Two samples are significantly different when p<0.05.
(1.31 MB TIF)

Table S5 Probability values obtained after a student's t-test on qPCR results shown in figure 4. Two samples are significantly different when p<0.05.
(0.76 MB TIF)

Table S6 List of primers used in this study.
(1.12 MB TIF)
