# Supplementary figures and images for: Parental Genome Dosage Imbalance Deregulates Imprinting in Arabidopsis
Source: PLoS Genet. 2010 Mar 19;6(3):e1000885. doi: 10.1371/journal.pgen.1000885 (PMC2841625; doi:10.1371/journal.pgen.1000885)

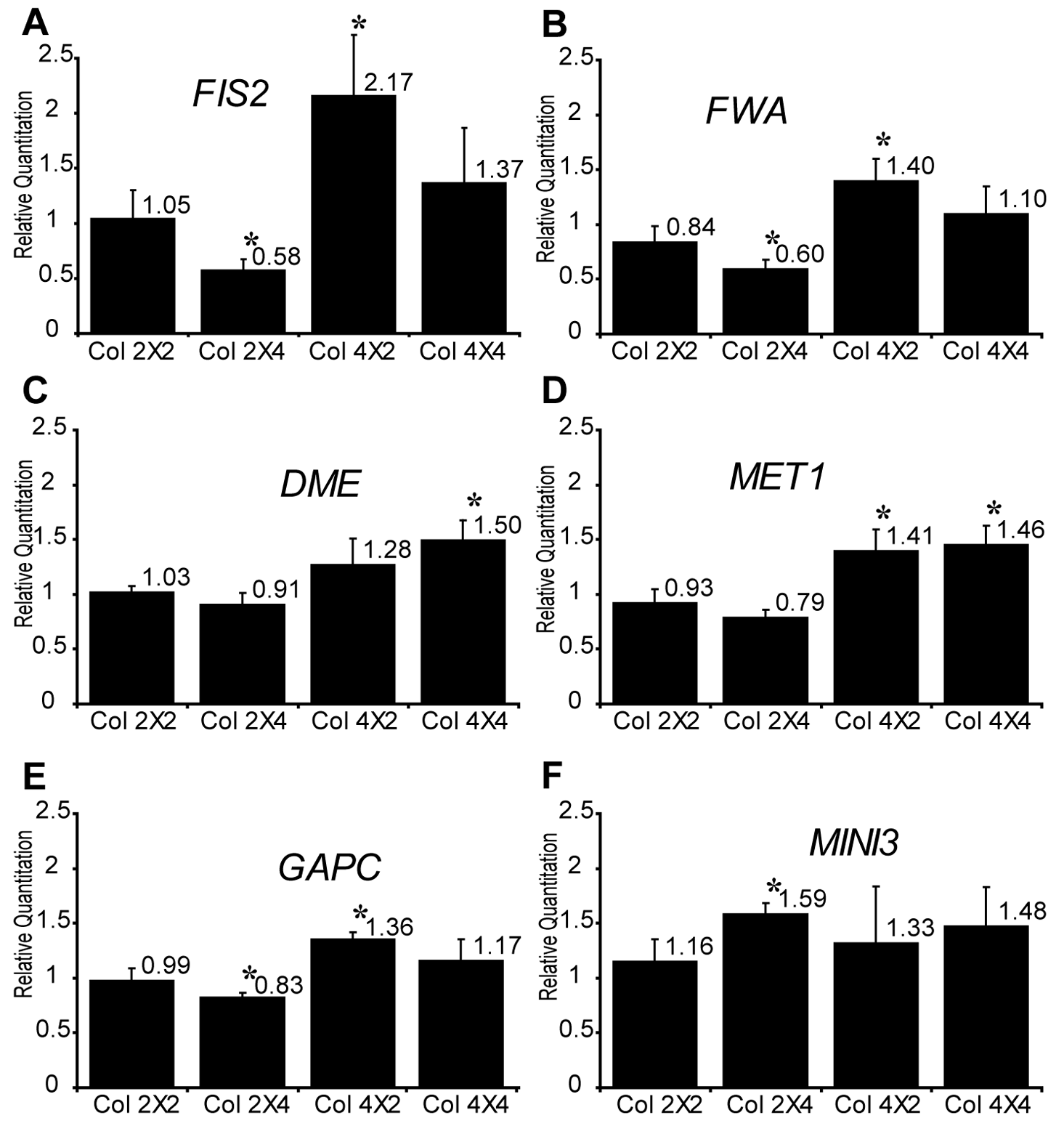

Supplement: Figure S1 — Effects of interploid crosses on the expression of DNA methylation dependent imprinted genes. (A-D) Quantitative PCR measurements of FIS2 (A), FWA (B), DME (C), MET1 (D), GAPC (E), and MINI3 (F) mRNAs were performed on total mRNAs extracted from siliques produced by crosses between diploid and tetraploid parents (2 DAP, Col ecotype). Each point represents the average RQ value obtained for four independent biological samples (Table S3). Error bars represent the standard deviation. * represents p<0.05 of t-test using Col 2X2 as a reference, p values can be found in Table S4. (0.89 MB TIF) [file pgen.1000885.s001.tif]

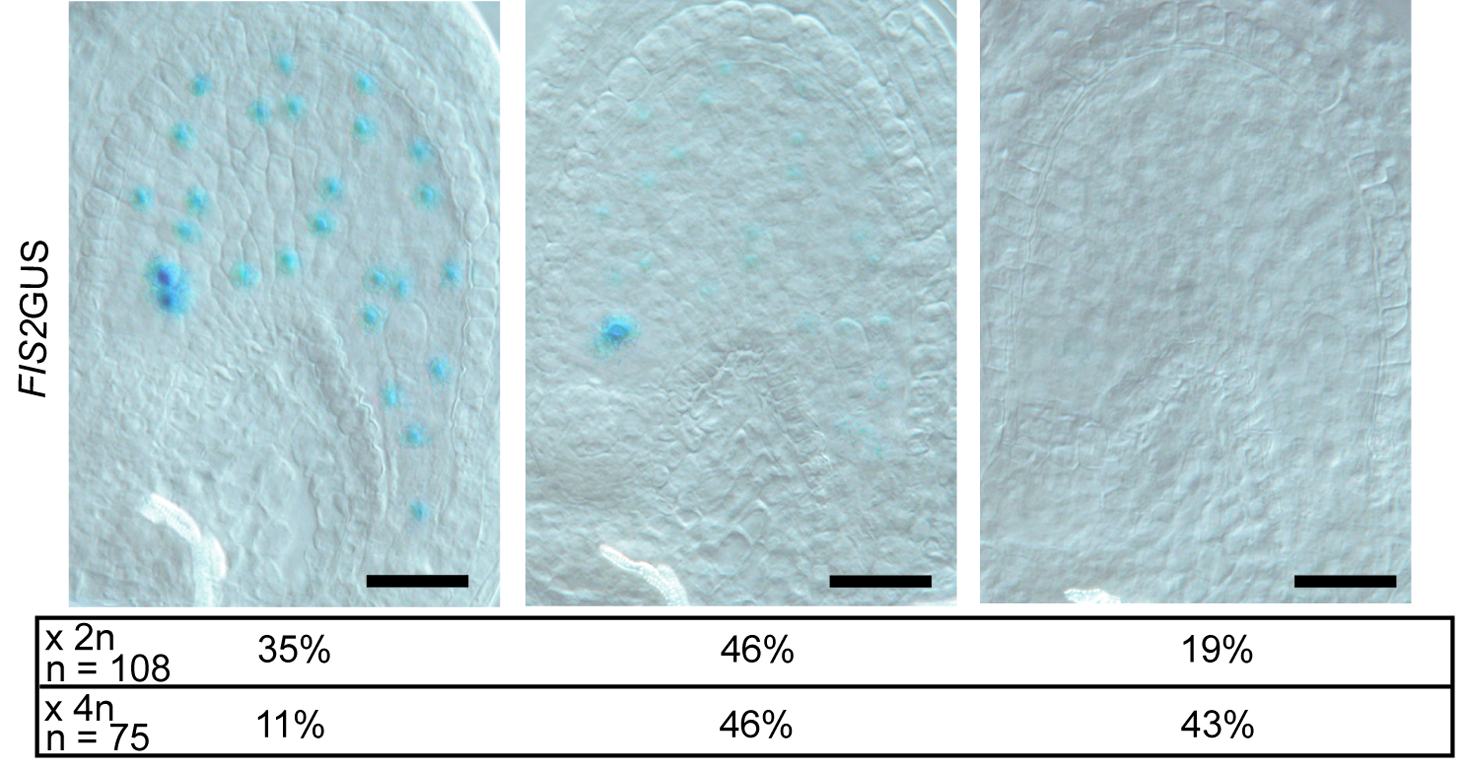

Supplement: Figure S2 — Effects of interploid crosses on the expression FIS2 transgenes. Effect of an increased maternal dosage on expression of transcriptional reporter pFIS2-GUS expression at 1.5 DAP. Staining was stopped before signal saturation and three classes of seeds were distinguished on the basis of the intensity of signal. The percentage of each class in crosses between ovules of the marker line and wild-type pollen from diploid or tetraploid plants is indicated below each corresponding micrograph. Scale bars correspond to 25 µm. (2.58 MB TIF) [file pgen.1000885.s002.tif]

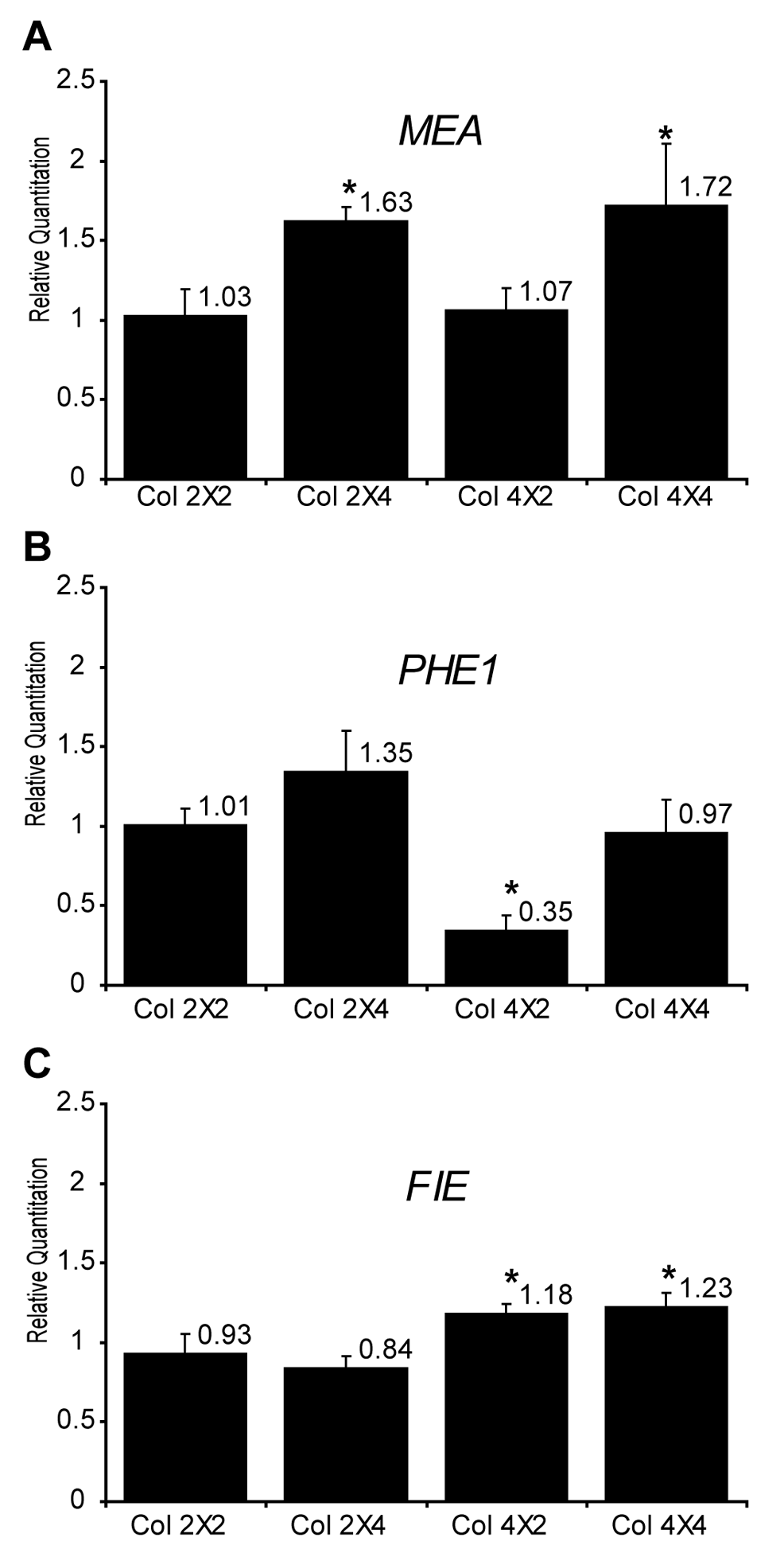

Supplement: Figure S3 — Effects of interploid crosses on the expression of genes imprinted by Polycomb group activity. Quantitative PCR measurements of MEA (A), PHE1 (B), and FIE (C) mRNAs were performed on total mRNAs extracted from siliques produced by crosses between diploid and tetraploid parents (2 DAP, Col ecotype). Each point represents the average RQ value obtained for four independent biological samples (Table S3). Error bars represent the standard deviation. * represents p<0.05 of t-test using Col2X2 as a reference, p values can be found in Table S4. (0.75 MB TIF) [file pgen.1000885.s003.tif]

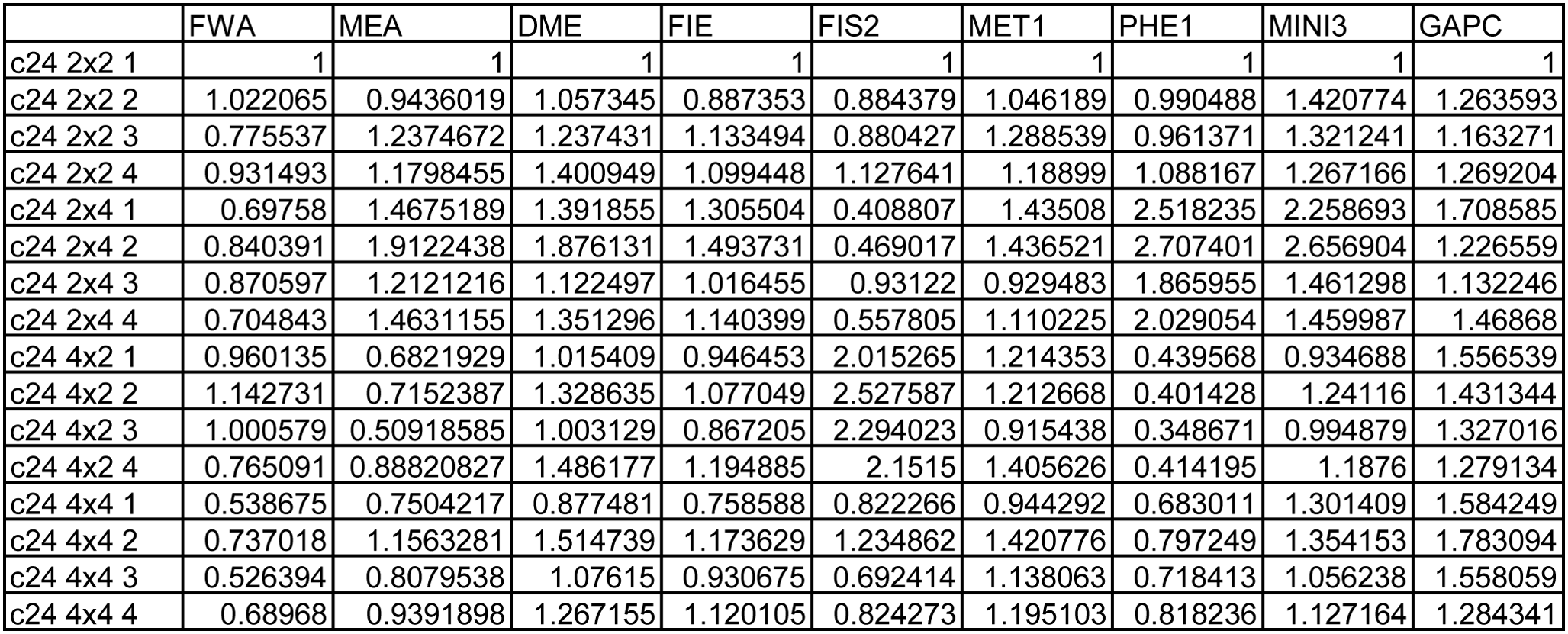

Supplement: Table S1 — RQ value of the C24 experiment after normalisation with Act11. C24 2nX2n sample 1 was normalised to 1. Each RQ value in this table corresponds to the average RQ value of 3 technical replicates. (1.32 MB TIF) [file pgen.1000885.s004.tif]

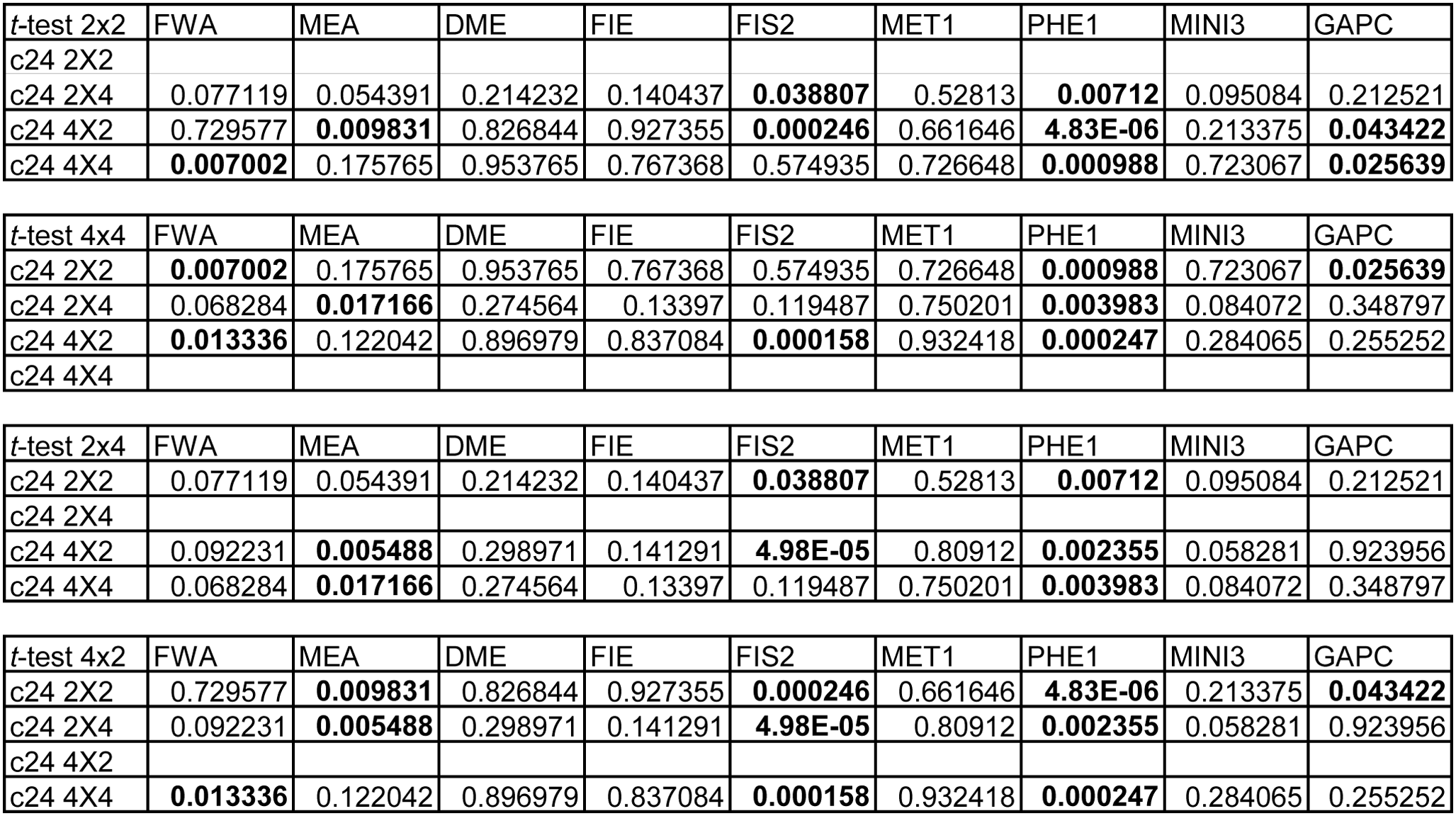

Supplement: Table S2 — Probability values obtained after a student's t-test on C24 sets of crosses from Figure 1 and Figure 3. Two samples are significantly different when p<0.05. (1.31 MB TIF) [file pgen.1000885.s005.tif]

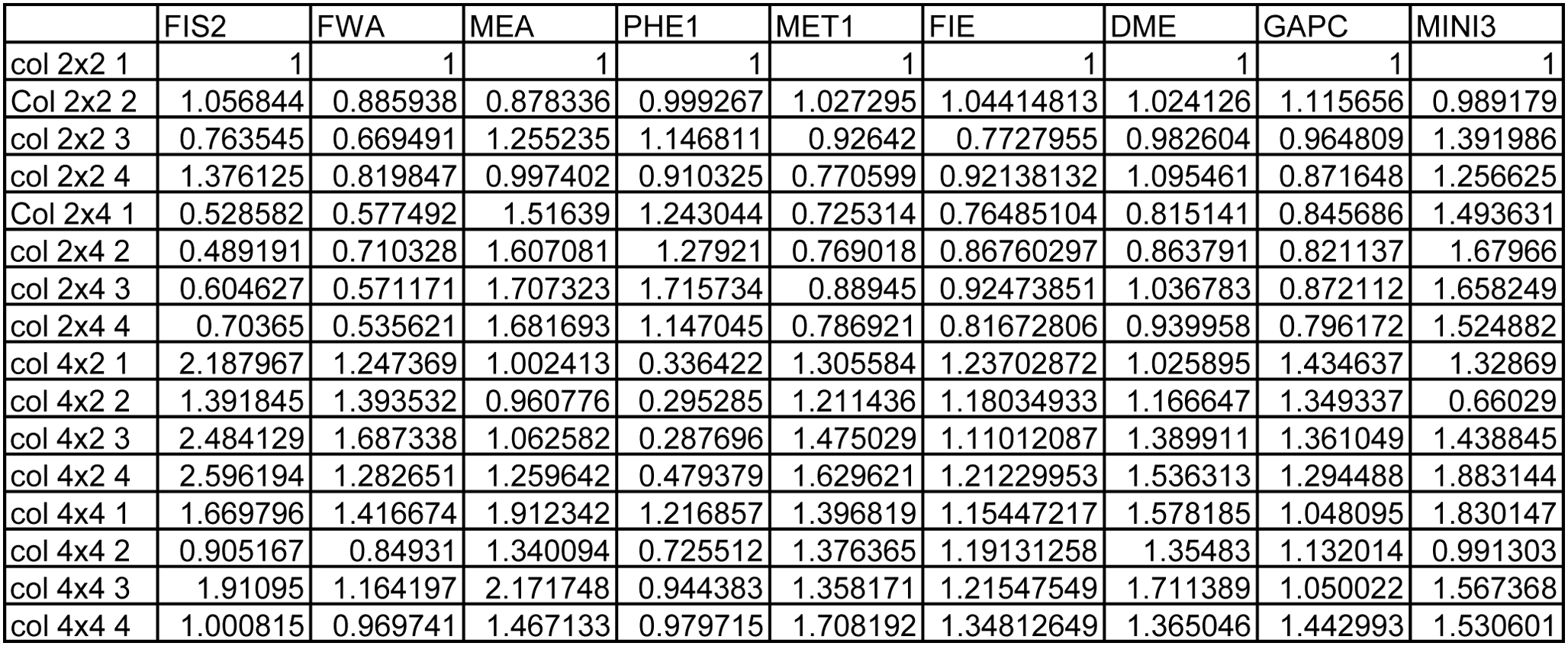

Supplement: Table S3 — RQ value of the Columbia experiment after normalisation with Act11. Col 2nX2n sample 1 was normalised to 1. Each RQ value in this table corresponds to the average RQ value of 3 technical replicates. (1.34 MB TIF) [file pgen.1000885.s006.tif]

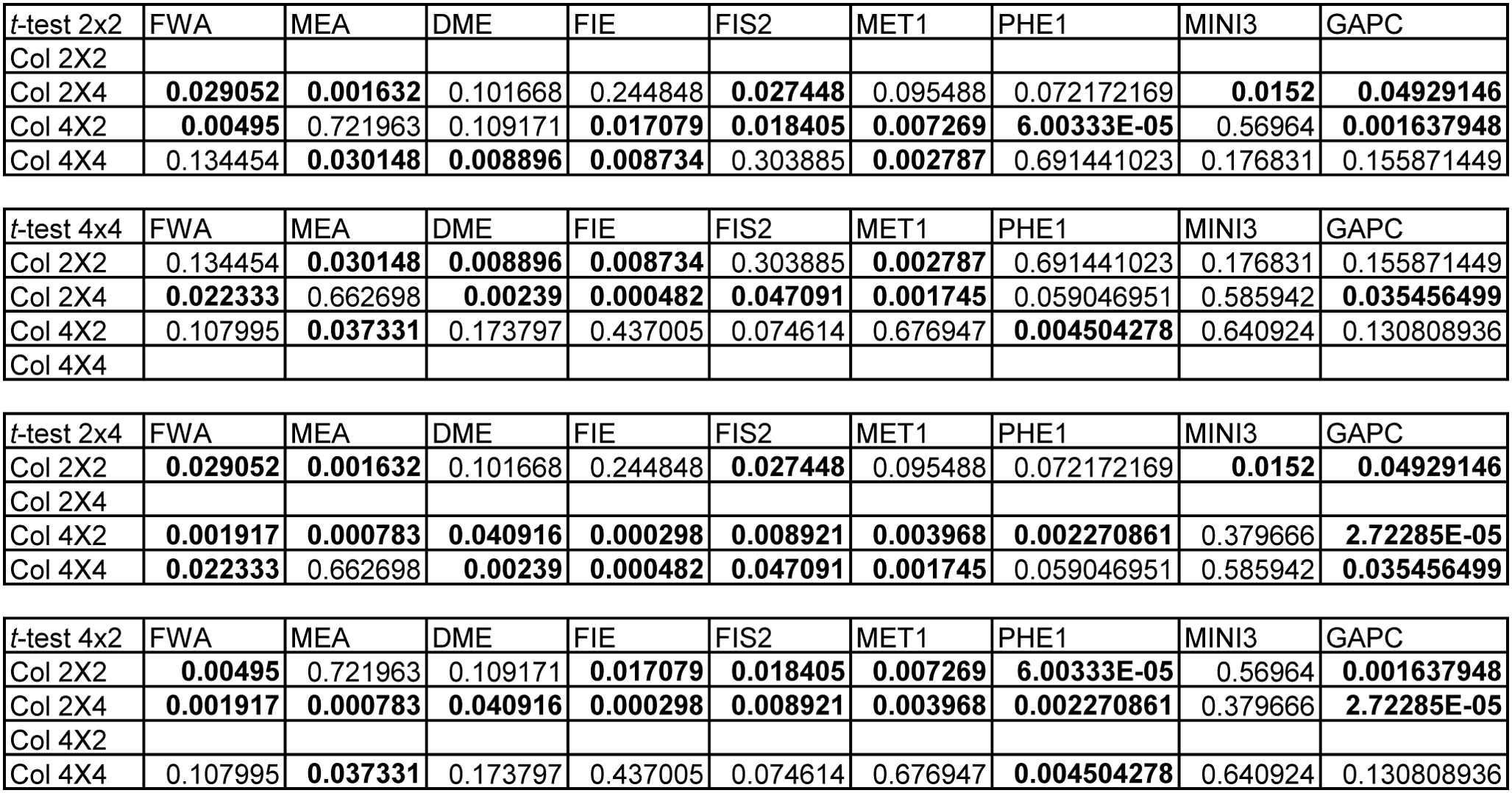

Supplement: Table S4 — Probability values obtained after a student's t-test on Col sets of crosses from Figure S1 and Figure S3. Two samples are significantly different when p<0.05. (1.31 MB TIF) [file pgen.1000885.s007.tif]

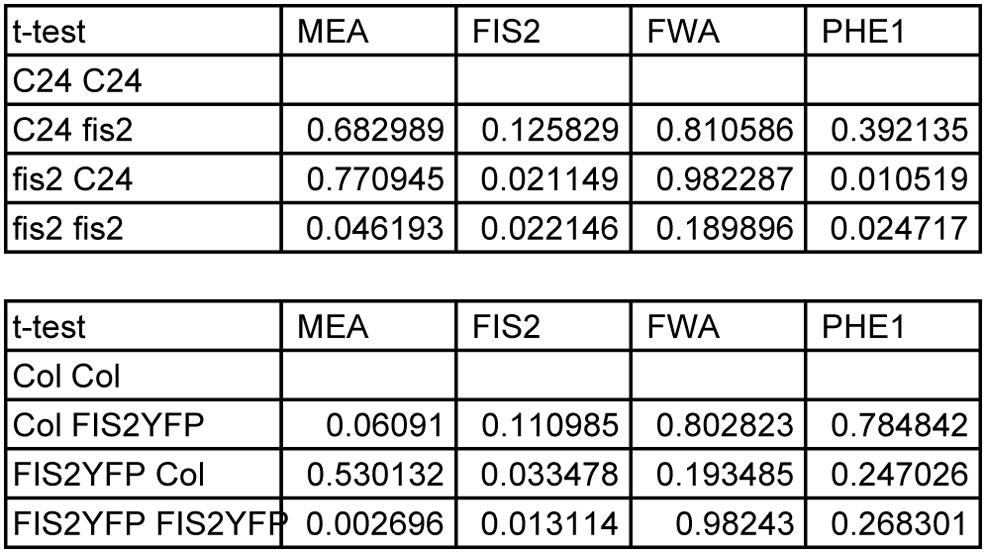

Supplement: Table S5 — Probability values obtained after a student's t-test on qPCR results shown in figure 4. Two samples are significantly different when p<0.05. (0.76 MB TIF) [file pgen.1000885.s008.tif]

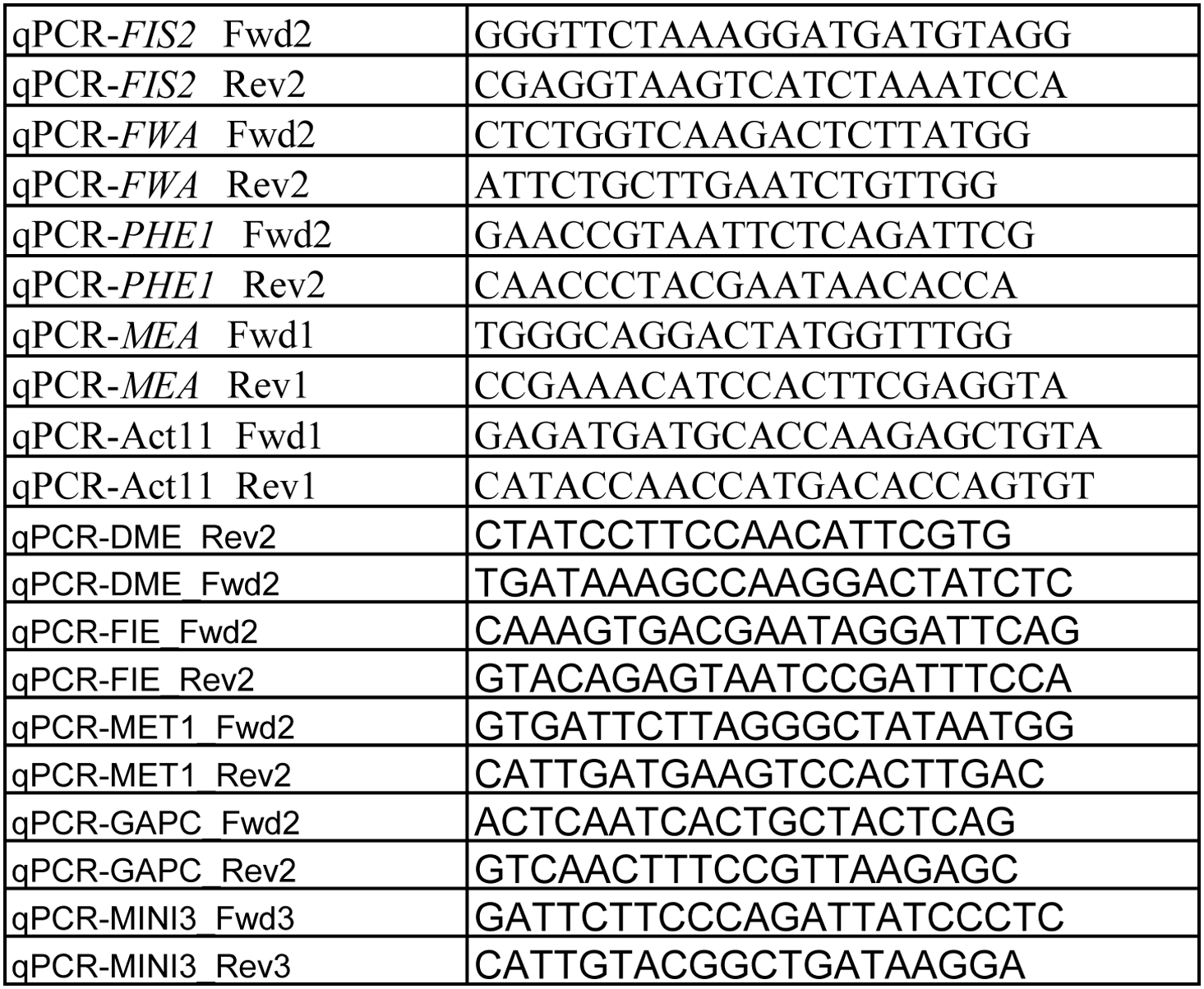

Supplement: Table S6 — List of primers used in this study. (1.12 MB TIF) [file pgen.1000885.s009.tif]
